# Melatonin and Vitamins as Protectors against the Reproductive Toxicity of Bisphenols: Which Is the Most Effective? A Systematic Review and Meta-Analysis

**DOI:** 10.3390/ijms241914930

**Published:** 2023-10-05

**Authors:** Sheila I. Peña-Corona, Juan I. Chávez-Corona, Luis E. Pérez-Caltzontzin, Dinorah Vargas-Estrada, C. Adriana Mendoza-Rodríguez, Edgar Ramos-Martínez, Jose L. Cerbón-Gutiérrez, José A. Herrera-Barragán, David Quintanar-Guerrero, Gerardo Leyva-Gómez

**Affiliations:** 1 Departamento de Farmacia, Facultad de Química, Universidad Nacional Autónoma de México, Ciudad de México 04510, Mexico; sheila.ipc@live.com (S.I.P.-C.); lepcqfb@outlook.com (L.E.P.-C.); 2 Laboratorio de Investigación y Posgrado en Tecnología Farmacéutica, Universidad Nacional Autónoma de México-FESC, Campus 1, Cuautitlán Izcalli 54714, Mexico; juan.isaac.chavez@gmail.com (J.I.C.-C.); quintana@unam.mx (D.Q.-G.); 3 Departamento de Fisiología y Farmacología, Facultad de Medicina Veterinaria y Zootecnia, Universidad Nacional Autónoma de México, Ciudad de México 04510, Mexico; dinorahvestrada@fmvz.unam.mx; 4 Departamento de Biología, Facultad de Química, Universidad Nacional Autónoma de México, Ciudad de México 04510, Mexico; adrimed@yahoo.com (C.A.M.-R.); edgargus2@gmail.com (E.R.-M.); 5 Escuela de Ciencias, Universidad Autónoma Benito Juárez de Oaxaca, Oaxaca 04510, Mexico; 6 Departamento de Reproducción, Facultad de Medicina Veterinaria y Zootecnia, Universidad Nacional Autónoma de México, Ciudad de México 04510, Mexico; cerron@unam.mx; 7 Departamento de Producción Agricola y Animal, Universidad Autónoma Metropolitana Unidad Xochimilco, Ciudad de México 04960, Mexico; jherrerab@correo.xoc.uam.mx

**Keywords:** hormone receptors, endocrine disruptors, reproduction, molecular docking, sperm

## Abstract

Bisphenols such as bisphenol A (BPA), S (BPS), C (BPC), F (BPF), AF (BPAF), tetrabromobisphenol, nonylphenol, and octylphenol are plasticizers used worldwide to manufacture daily-use articles. Exposure to these compounds is related to many pathologies of public health importance, such as infertility. Using a protector compound against the reproductive toxicological effects of bisphenols is of scientific interest. Melatonin and vitamins have been tested, but the results are not conclusive. To this end, this systematic review and meta-analysis compared the response of reproductive variables to melatonin and vitamin administration as protectors against damage caused by bisphenols. We search for controlled studies of male rats exposed to bisphenols to induce alterations in reproduction, with at least one intervention group receiving melatonin or vitamins (B, C, or E). Also, molecular docking simulations were performed between the androgen (AR) and estrogen receptors (ER), melatonin, and vitamins. About 1234 records were initially found; finally, 13 studies were qualified for review and meta-analysis. Melatonin plus bisphenol improves sperm concentration and viability of sperm and increases testosterone serum levels compared with control groups; however, groups receiving vitamins plus bisphenols had lower sperm concentration, total testis weight, and testosterone serum levels than the control. In the docking analysis, vitamin E had the highest negative MolDock score, representing the best binding affinity with AR and ER, compared with other vitamins and melatonin in the docking. Our findings suggest that vitamins could act as an endocrine disruptor, and melatonin is most effective in protecting against the toxic effects of bisphenols.

## 1. Introduction

In recent years, scientific interest has increased regarding the impact of the toxicity of industrial contaminants on human health [1,2,3]. In recent decades, human and animal exposure to bisphenols, such as bisphenol A (BPA), S (BPS), C (BPC), F (BPF), AF (BPAF), tetrabromobisphenol (TBBPA), nonylphenol, and octylphenol, through ingestion, inhalation, and skin contact and the release of bisphenols into the environment have been growing [4,5]. Bisphenols are detected in water, food, and indoor dust [6] and have a short environmental half-life (2.5–4 days). Their pseudo-persistent disposition comes from the continuous manufacture and release of plastic containers with bisphenols in their composition [7]. For example, the annual bisphenol A (BPA) production exceeds 3.1 million tons [8]. So, bisphenols are ubiquitous compounds.

Humanity has used these compounds for many decades. These chemicals are formed by merging phenol with acetone to generate BPA or with hexafluoroacetone to synthesize bisphenol AF (BPAF), or with formaldehyde to produce bisphenol-F (BPF), or with sulfur trioxide to manufacture bisphenol-S (BPS). BPA and its analogs have high resistance to breaking, temperature, and electricity, so it has become widely used as a crosslinking chemical in producing polycarbonate plastic and epoxy resin used in daily consumer products [9,10].

Intensive experimental, epidemiological, and clinical investigations have focused on bisphenols, especially in BPA, which was synthesized initially as a synthetic estrogen [9,11]. The United States Environmental Protection Agency (EPA) has classified BPA as the third highest-priority chemical regarding its toxic profile [12]. Analogs such as BPS, BPAF, and BPF are commonly used to avoid BPA use in “BPA-free” products [13]. However, associations between these chemical exposures and several major diseases, such as behavioral problems, cardiovascular diseases, diabetes, cancer, and endocrine disorders, such as impairment in fertility, spermatogenesis, sperm motility, and concentration, have been described [14,15]. Therefore, bisphenols are considered endocrine-disrupting compounds (EDCs). 

In the reproduction field, the balance of oxidant species is essential because it relates to the production, maturation, morphological reshaping of spermatozoa, and fertilization [16]. Spermatozoa are significantly susceptible to oxidation because of the abundance of unsaturated fatty acids in the membrane, the deficiency of DNA repair mechanisms, and the lack of cytoplasmic antioxidant enzymes [17,18]. Thus, any compound that alters the balance of oxidant species will affect sperm characteristics.

The mode of action of bisphenols involves several cell pathways. They associate with membrane and nuclear estrogen receptors (ERs) α and β, androgen receptor (AR) [19], orphan receptors such as estrogen-related receptor γ [20] and aryl hydrocarbon receptor [21] and produce an imbalance between the cells’ free radical levels and the antioxidant defensive system [12,22,23]. The peroxidation of unsaturated fatty acids in the plasma membrane modifies sperm membrane fluidity and morphology [24]. Therefore, antioxidants could protect against the toxic effects of bisphenols. 

Despite the available information about the toxic effects of bisphenols, these chemicals are still in use as plasticizers. So, in recent years, research has focused on studying biomolecules with antioxidant properties, with the potential to protect or reverse the toxic effect produced by industrial contaminants [2]. In recent years, the antioxidant properties of melatonin [24], biopolymers [25], plants or their extracts [26,27,28], selenium, zinc [29,30], and vitamins (E (α-tocopherol), C, and B) have been tested [31,32,33].

In this regard, the main studied compounds are vitamins and melatonin. Vitamins improve hormonal and histological parameters, sperm volume, motility, concentration, and viability in rats with bisphenol-induced reproductive disorders [4,34]. In adult male Wistar rats, administration of 4 mg/100 g bw of Vitamin E (VitE) for three months protects against the harmful effects in reproductive parameters produced by BPA treatment (5, 50, 100 μg/100 g bw/d) [31]. VitC benefits sperm characteristics, destroying free radicals generated by environmental pollution and cellular metabolism, which would otherwise produce oxidative DNA damage [4]. In 2019, Gules et al. performed the first study concerning the antioxidant effect of VitB9 (folic acid) as a protector against BPA damage [32]. Melatonin administration reduces reproductive toxicity and protects the testis against bisphenol treatment [24,35,36,37]. However, to our knowledge, no studies statistically compare the protective effects and describe the most effective compounds against the toxic consequences of bisphenols.

Through a systematic review and meta-analysis of data, this paper aimed to compare the response in reproductive variables of melatonin and vitamin administration in combination with bisphenols as protection against reproductive damage. In addition, we used molecular docking between the androgen receptor (AR) and ER with melatonin and vitamins as ligands to determine the probability of a stable union with the receptors and elucidate their possible involvement as endocrine disruptors.

## 2. Results

### 2.1. Selection and Identification of Studies

**Figure 1:**
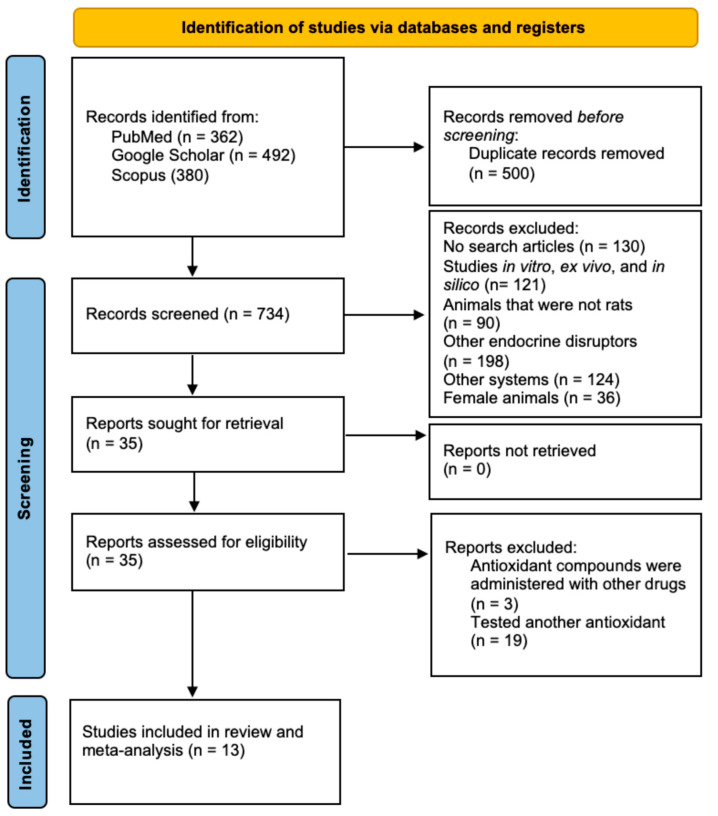
Flow diagram of the literature search and selection process.

We followed the PRISMA statement [38] and found 1234 articles. After duplicates were removed, 734 studies were screened according to the inclusion criteria, and 13 studies were eligible to be included in the narrative review and meta-analysis. The PRISMA flow diagram of the literature search is shown in Figure 1.

### 2.2. Study Characteristics

We found 13 relevant studies published between 2003 and 2021; 4 used treatments with melatonin alone [24,36,37,39], and 9 explored various vitamins. VitE was used in five studies [31,40,41,42,43], one study applied VitE and melatonin as independent treatments in the same study [44], two studies worked with vitamin C (VitC) [4,33], and one study used vitamin B9 (VitB9) (folic acid) [32] (Table 1). The studies used Wistar and Sprague Dawley rats, weighing 150 and 400 g and aged 6 to 16 weeks old; the age at sample collection was 11 to 24 weeks old. Around 492 animals were utilized for the experiments reported in the articles (Table 1). 

Studies evaluated the toxic effect of BPA; additionally, Aydoğan et al. considered nonylphenol and octylphenol [33]. Chitra et al. [4] assessed the smallest BPA dose of 0.0002 mg/kg bw/d, and the highest amount tested was 325 mg/kg/d in the study of Omran et al. [43]. The shortest BPA exposure span was 10 days [39], and the longest was 90 days [31]. Except for Rashad et al. [44], who administered the BPA intraperitoneally (i.p.), all other studies administered it to the animals orally (p.o.). On the other hand, almost all studies administered melatonin or vitamins p.o., except Olukole et al. (2018) [37] and Rashad et al. (2021) [44], who administered melatonin i.p. (Table 2). 

### 2.3. Meta-Analysis 

#### 2.3.1. Final Body Weight 

Not enough studies used melatonin to analyze final animal weight. However, in three studies that employed vitamins [32,33,40], our results suggest that the administration of bisphenols plus vitamins decreased the final body weight compared with the control group (*p* < 0.05). No differences were obtained in other comparisons, which suggested a synergic effect between the harmful effects of bisphenols and vitamins (Table 3).

#### 2.3.2. Testosterone 

Studies show that bisphenols decrease testosterone serum levels (*p* < 0.05, Table 3), but adding melatonin [36,37,44] or vitamins [31,32,40,41,43,44] significantly ameliorated the bisphenol-induced decrease in testosterone serum levels (*p* < 0.05). Even melatonin prevents the decrease in serum testosterone levels since there were no significant differences between BPA plus melatonin groups and control groups (*p* > 0.05). However, administering bisphenols plus vitamins significantly decreases testosterone levels compared to the control group (*p* < 0.05, Table 3).

#### 2.3.3. Sperm Characteristics 

Bisphenol treatment decreased sperm concentration compared with the control group in studies performed with melatonin and vitamins (*p* < 0.05). However, when bisphenol was combined with melatonin [24,36,39,44] or vitamins [4,31,33,40,41,44], the sperm concentration was improved compared to that of the bisphenol group (*p* < 0.05) (Table 4). The administration of bisphenol and melatonin was not significantly different from the control (*p* > 0.05), so melatonin could inhibit the reduction in sperm concentration produced by BPA (Table 4), while vitamins in combination with bisphenol may decrease sperm concentration compared to the control (*p* < 0.05, Table 3).

Bisphenol lowered sperm motility in studies performed with melatonin [24,36,44] and vitamins [4,44] compared to a control (*p* < 0.05, Table 3). There were no differences between the bisphenol plus melatonin administration groups and the bisphenol or control groups (*p* > 0.05, Table 3). Vitamin administration with bisphenols improved sperm motility compared to bisphenols alone (*p* < 0.05, Table 3); additionally, there were no differences when the sperm motility was compared with the control (*p* > 0.05, Table 3), which indicated that vitamins efficiently inhibited the BPA-induced decrease in sperm motility.

Bisphenol decreased sperm viability compared to the control group in studies performed with melatonin (*p* < 0.05, Table 3) [24,36]. Exposure to melatonin with bisphenols improved sperm viability, compared to bisphenols alone (*p* > 0.05, Table 3). Bisphenol plus melatonin significantly decreased sperm viability compared to the control group. There were no differences between the bisphenol and control groups and between the bisphenols plus vitamins and bisphenol groups (*p* > 0.05, Table 3) [4,32]. Administration of bisphenols plus vitamins significantly decreased sperm viability compared to a control, which suggested a synergic effect between the harmful effect of bisphenols and vitamins.

Not enough studies used melatonin to analyze abnormal sperm morphology (head, neck, and tail). In the bisphenol plus vitamin administration groups, there was a significant increase in abnormal sperm morphology compared to control groups (*p* < 0.05) [32,33,44], and there were no differences compared to the bisphenol group (*p* > 0.05, Table 3), which indicated that vitamins could not avoid the detrimental effects of BPA on sperm morphology. In the morphology of the head [32,33], neck [32,33], and tail of sperm [32,33], there were no differences between the bisphenol and control groups (*p* > 0.05, Table 3). Additionally, there were no differences between the bisphenols plus vitamins and bisphenol groups. However, bisphenols plus vitamins increased the head and neck sperm abnormalities compared to the control group (*p* < 0.05, Table 3), which suggested a synergic effect between the harmful effects of bisphenols and vitamins.

#### 2.3.4. Testis Weights

Not enough studies used melatonin to analyze the weight of the testis; however, studies that used vitamins reported a significant decrease [31,32,40] in the bisphenol and the bisphenol plus vitamin groups compared to control (*p* < 0.05). Administering bisphenols plus vitamins improved the total testis weight compared with the bisphenol group (*p* < 0.05). The data from the right [32,33] and left [32,33] testes illustrated that the administration of bisphenols plus vitamins significantly decreased the weight of the right and left testes compared with the control (*p* < 0.05, Table 3), which suggested a synergic effect between the harmful effects of bisphenols and vitamins.

In summary, administering melatonin and vitamins in addition to bisphenol treatment improved sperm concentration and testosterone serum levels compared with bisphenol-alone groups (*p* < 0.05). Melatonin administration in animals treated with bisphenols protected the reproductive variables against the harmful bisphenol effects, making the size of these variables similar to those found in the control group because there were no significant differences in testosterone serum levels, sperm concentration, and motility between bisphenols plus melatonin and the control groups (*p* > 0.05). Rats given bisphenol and vitamins had lower sperm concentration, total testis weight, and testosterone serum levels than the control (*p* < 0.05). Even more, the administration of vitamins in rats treated with bisphenols provoked a further increase in the damage provoked by bisphenol in the final body and right and left testis weight, in the abnormalities of sperm morphology (head and neck), and in sperm viability compared to the control, which suggested a synergic effect between the harmful effects of bisphenols and vitamins (Table 3). 

#### 2.3.5. Outliers

There was no indication of outliers in the context of this model in the administration of bisphenols plus melatonin or vitamins compared with the control. According to Cook’s distances, none of the studies could be considered overly influential.

### 2.4. Risk of Bias Assessment

**Figure 2:**
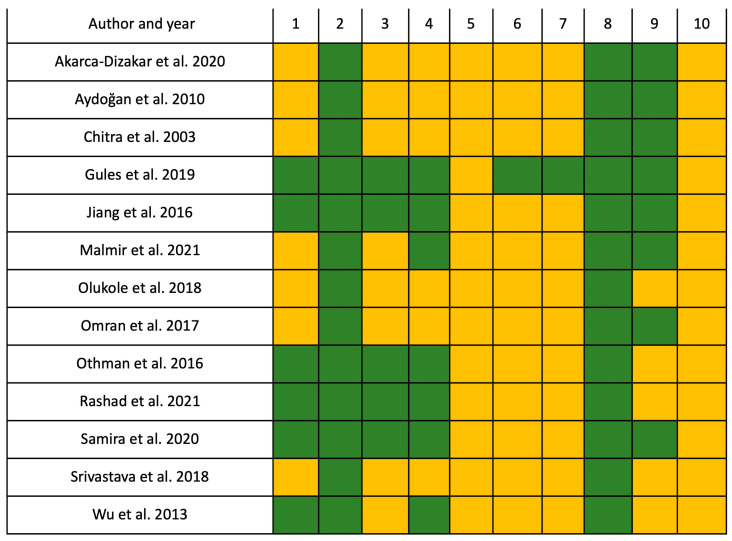
Assessment using SYRCLE’s risk of bias tool. Articles are categorized by color (green indicates low risk, and yellow color designates unclear) using SYRCLE’s risk of bias tool. 1: selection bias, sequence generation; 2: selection bias, baseline characteristics; 3: selection bias, allocation concealment; 4: performance bias, random housing; 5: performance bias, blinding; 6: detection bias, random outcome assessment; 7: detection bias, blinding; 8: attrition bias, incomplete outcome data; 9: reporting bias, selective outcome reporting; 10: other sources of bias. Akarca-Dizakar et al. 2020 [24], Aydoğan et al. 2010 [33], Chitra et al. 2003 [4], Gules et al. 2019 [32], Jiang et al. 2016 [41], Malmir et al. 2021 [42], Olukole et al. 2018 [37], Omran et al. 2017 [43], Othman et al. 2016 [36], Rashad et al. 2021 [44], Samira et al. 2020 [40], Srivastava et al. 2018 [31], Wu et al. 2013 [39].

According to the SYRCLE’s risk of bias results, 40.77% (54/130) of all items were classified as low-risk, and 59.23% (76/130) were unclear. For selection bias (items 1, 2, and 3), 61.54% (24/39) were low-risk, and 38.46% (15/39) were unclear. Regarding the performance bias (items 4 and 5), which was the most affected bias, 26.92% (7/26) were low-risk, and 73.08% (19/26) were unclear. For detection bias (items 6 and 7), 7.7% (2/26) were low-risk, while 92.3% (24/26) were unclear. For attrition bias (item: 8), which was the least affected bias, 92.3% (12/13) were low-risk, and 7.7% (1/13) were unclear. For reporting bias (item: 9), 61.53% (8/13) were low-risk, and 38.46% (5/13) were unclear. Finally, for other biases, 100% (13/13) were unclear (Figure 2).

### 2.5. Molecular Modeling of Melatonin and Vitamin Interactions with Estrogen and Testosterone Receptors

**Figure 3:**
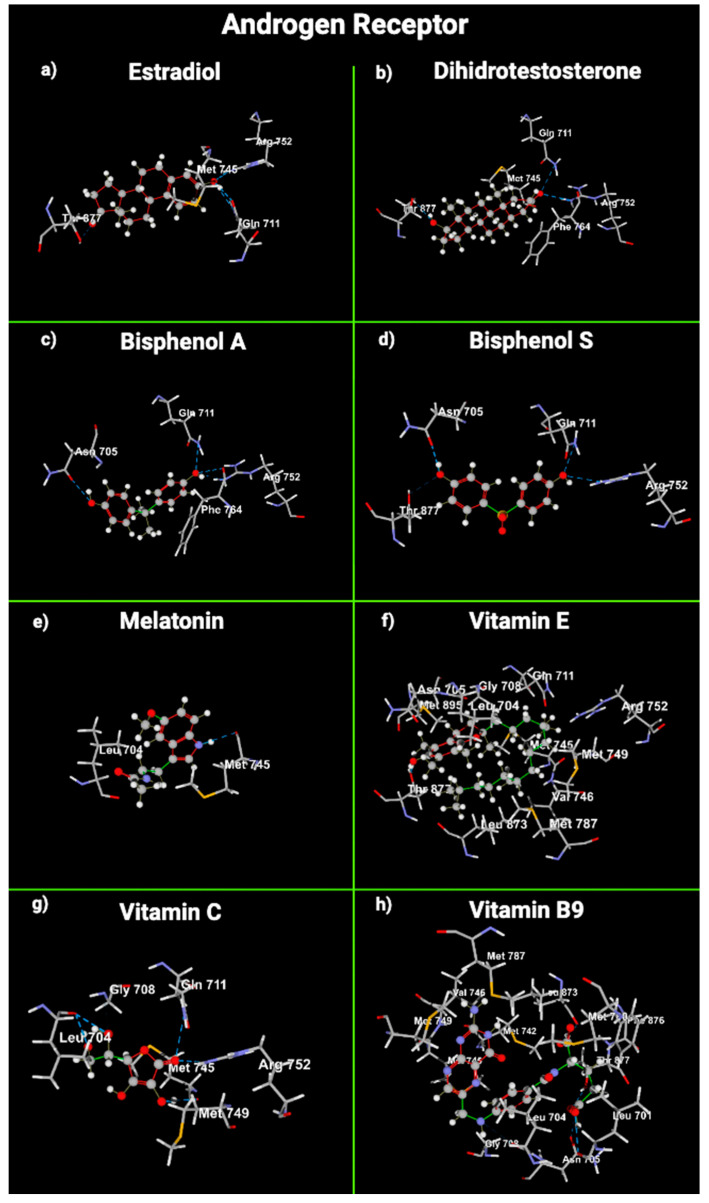
Cross-sectional view of androgen receptor’s amino acids that interact with (**a**) estradiol, (**b**) dihydrotestosterone, (**c**) bisphenol A, (**d**) bisphenol S, (**e**) melatonin, (**f**) vitamin E, (**g**) vitamin C, and (**h**) vitamin B9. Gray: carbon atoms; white: hydrogen atoms; red: oxygen atoms; blue/purple: nitrogen atoms; yellow: sulfur atoms.

**Figure 4:**
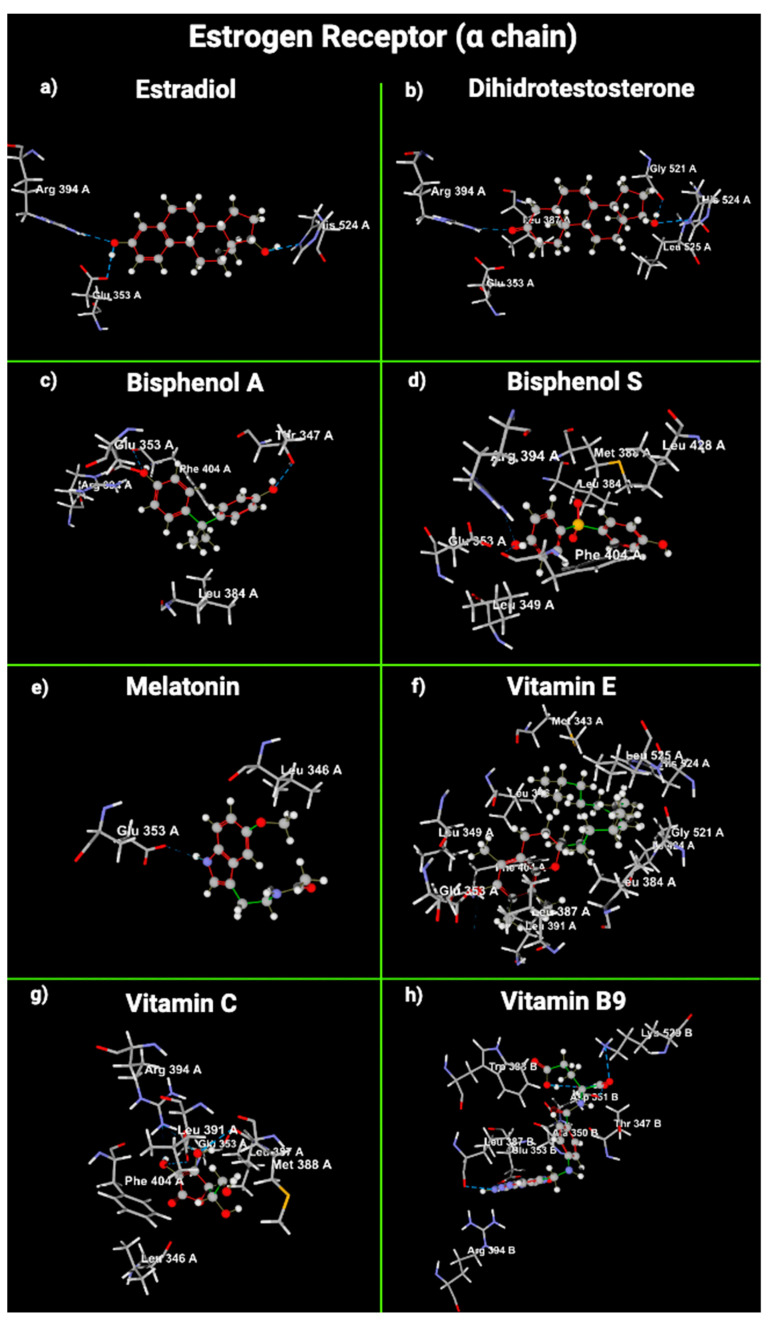
Cross-sectional view of the estrogen receptor α amino acids that interact with (**a**) estradiol, (**b**) dihydrotestosterone, (**c**) bisphenol A, (**d**) bisphenol S, (**e**) melatonin, (**f**) vitamin E, (**g**) vitamin C, and (**h**) vitamin B9. Gray: carbon atoms; white: hydrogen atoms; red: oxygen atoms; blue/purple: nitrogen atoms; yellow: sulfur atoms.

**Figure 5:**
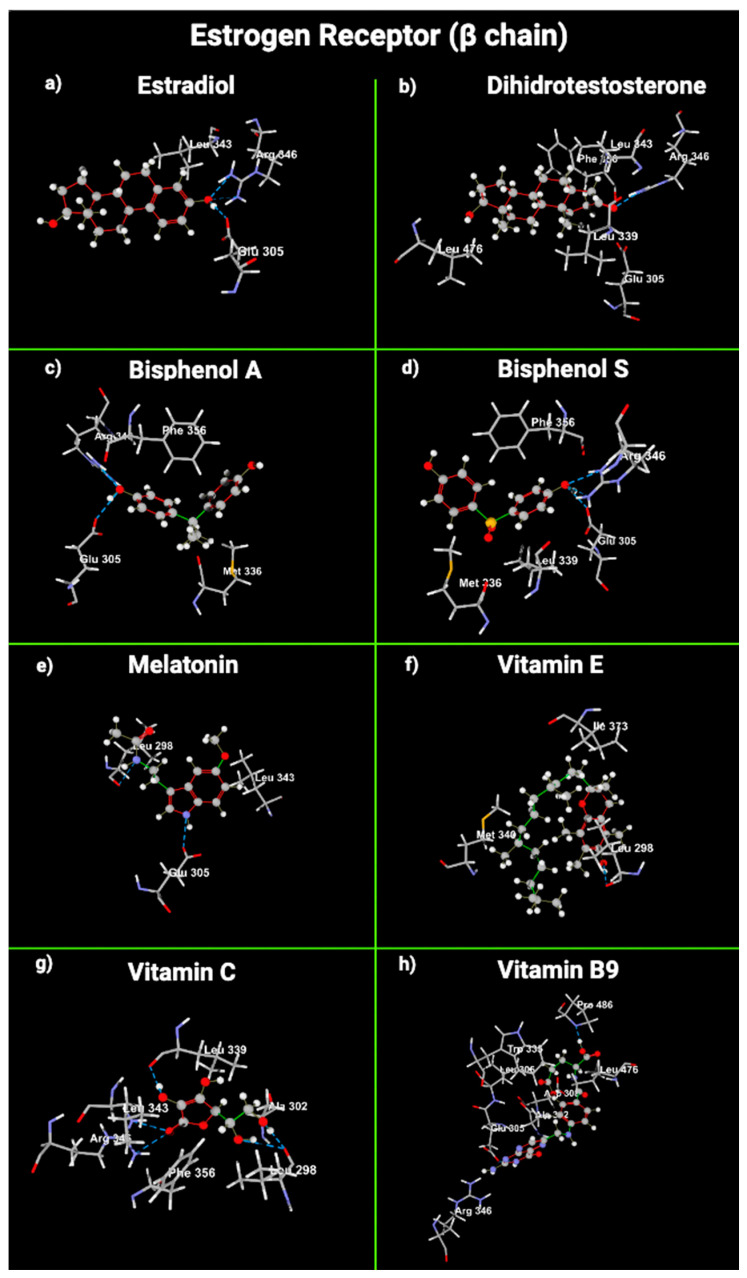
Cross-sectional view of the estrogen receptor β amino acids that interact with (**a**) estradiol, (**b**) dihydrotestosterone, (**c**) bisphenol A, (**d**) bisphenol S, (**e**) melatonin, (**f**) vitamin E, (**g**) vitamin C, and (**h**) vitamin B9. Gray: carbon atoms; white: hydrogen atoms; red: oxygen atoms; blue/purple: nitrogen atoms; yellow: sulfur atoms.

We used molecular docking to gain insight into the molecular interactions between the ligands E2, dihydrotestosterone (DHT), BPA, BPS, melatonin, VitE, VitC, and VitB9 and the union sites of AR, ERα, and ERβ (Figure 3, Figure 4 and Figure 5). 

DHT interacts with AR through hydrogen bonds with Arg 752, Thr 877, and Gln 711. These three amino acids also form a union between E2 and AR. VitC, BPA, and BPS used Arg 752 and Gln 711 to interact with AR. VitB9 and VitE used Thr 877. Melatonin did not share any amino acids with DHT in the union with AR. DHT sterically interacted with AR using Met 745 and Phe 764; E2 and VitB9 also used Met 745, and VitE presented the same steric interactions as DHT (Table 4).

Regarding hydrogen bond interactions between ligands and ERα, E2 interacts with it using Arg 394, His 524, and Glu 353. Melatonin does not interact with E2α. VitE interacts with ERα using Glu 353. VitC joins ERα with Arg 394 and Glu 353; VitB9 uses Glu 353. DHT used Arg 394 and His 524 to join with ERα, and BPA and BPS joined with ERα through Phe 404; this amino acid was not used in the interaction between E2 and ERα (Table 4). 

Concerning ERβ, E2 interacts with it through Arg 346 and Glu 260. Arg 346 is also used in the interaction between ERβ and DHT, BPA, BPS, and VitC (Table 4). Melatonin and VitB9 did not use the amino acids involved in the interaction between E2 and ERβ. The amino acids with steric interaction between AR and DHT were Met 745 and Phe 764. AR used Met 745 in the steric interaction with VitE and VitB9. E2α did not have any steric interaction with ERα. Regarding ERα, the amino acid that presented steric interactions with DHT, BPA, BPS, melatonin, VitE, and VitC was Glu 353. E2 formed steric interactions with Leu 343 of ERβ. Leu 343 was also used to join DHT, melatonin, and VitC (Table 4). 

**Figure 6:**
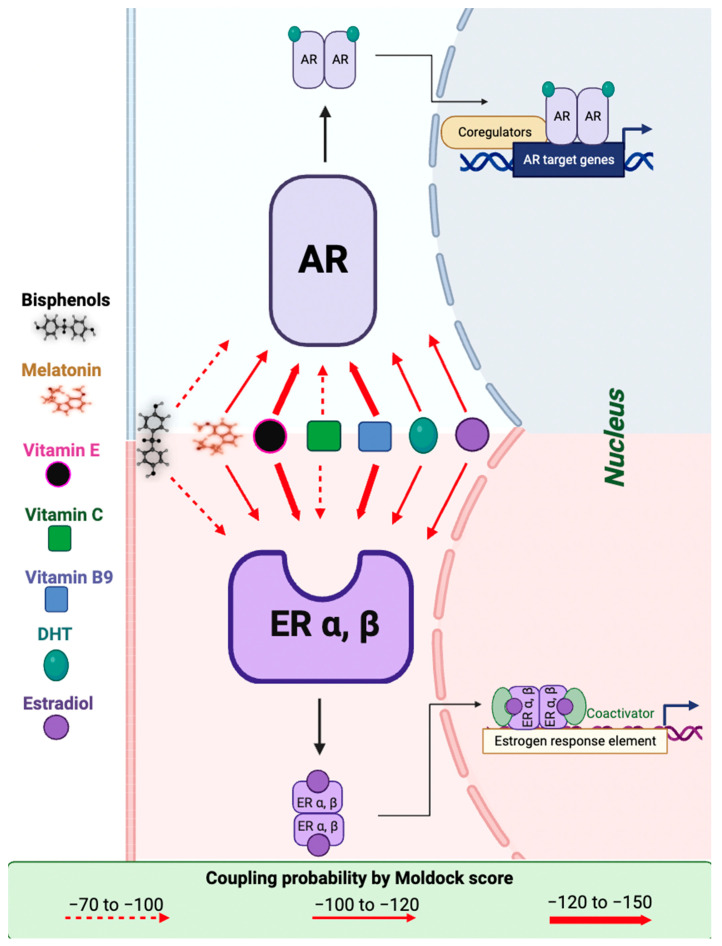
The schematic representation of interactions between estrogen and androgen receptors (ER and AR, respectively) and estrogen, bisphenols, dihydrotestosterone (DHT), estradiol, melatonin, vitamin E, vitamin C, and vitamin B9, according to the results of coupling probability determined by MolDock score (Molegro Virtual Docker 2007 version 6.0., Molegro Bioinformatics, Aarchus C, Denmark). Lower energy scores (most negative number) indicate more appropriate protein–ligand bindings than higher energy values. The dotted red arrow means a minor coupling probability with ER and AR (−70 to −100), and the thin red arrow means that the coupling probability with ER and AR is between −100 and −120 according to the MolDock score. A thick red arrow indicates that coupling probability according to the MolDock score is higher than that for other ligands.

Our MolDock score results were similar between the ligands and receptors. The lowest score (the highest negative number) was exhibited by VitE, followed by VitB9 (−120 to −150) with ERα, ERβ, and AR (thick red arrow, Figure 6). Melatonin, DHT, and estradiol presented similar MolDock score values (−100 to −120) in both receptors (thin red arrow); finally, bisphenols and VitC (dotted red arrow) show a MolDock score value between −70 and −100 for both receptors (Table 4, Figure 6).

## 3. Discussion

The use of melatonin and vitamins to protect organisms from bisphenol toxicity has been tested. However, the results have yet to be conclusive, and no evidence exists that indicates which is the most effective in protecting the reproductive system. Therefore, the present research aimed to compare the association between the response of reproductive variables to melatonin and vitamin administration as antioxidant compounds that could protect from the damage provoked by bisphenols. According to the results, compared with melatonin, vitamin intake is less effective in returning the reproductive variables affected by bisphenol treatment to levels similar to those of the animals in the control group. 

The mechanisms by which bisphenols exert their harmful effect as EDCs have not been thoroughly studied. However, several works proposed that bisphenols interfered with signaling pathways of the endocrine system, mimicked the action of estrogens [45,46], induced membrane lipid peroxidation, fragmented and disorganized spermatogenesis [47], and produced an imbalance of the cellular content of radical species and antioxidants leading to oxidative damage of cellular biomolecules [12,22,23]. As a result of our meta-analysis, as we expected, bisphenol, as an EDC [48], was found to produce harmful effects on reproductive variables [37], such as a significant decrease in testosterone serum concentration; sperm motility, viability, and concentration; and total testis weight and a significant increase in sperm abnormalities. Testosterone is crucial for maintaining spermatogenesis, sperm quality, and fertility [41,49]. So, it is probable that the decrease in testosterone levels could provoke these alterations in sperm. Consequently, morphologically abnormal sperm suggests that bisphenols administration prompts oxidative stress and reduces the oxidative phosphorylation of enzymes and ATP generation [50].

The male reproductive system has antioxidant defenses such as reduced glutathione (GSH), SOD, and CAT. Some exogenous antioxidants, such as vitamins, prevent oxidative damage in the testis instigated by reactive oxygen species (ROS) [33,51]. A balance between the concentration of ROS and antioxidants is essential in the testis for the adequate structure and function of sperm [52]. The increased oxidative stress damages sperm membranes, proteins, and DNA, reducing sperm viability [53]. Thus, using antioxidants, such as vitamins, could protect against the toxic effects of bisphenols [4]. VitE is an antioxidant with a lipophilic solid character. It can be found in Sertoli cells, pachytene spermatocytes, and round spermatids [54] and is essential for maintaining spermatogenesis in mammals [55]. In semen, VitE disrupts the activity of free radicals, prevents the creation of lipid peroxides in testicular microsomes and mitochondria, safeguards sperm against ROS damage [42], and is vital for preserving the mammalian spermatogenic process [56]. Some reports indicate that VitE and VitC protect against the damage produced by free radicals from EDC exposure in rodents [4,42]. VitB9 enhanced sperm concentration and motility in hypothyroid rats with testicular impairment [57]. Also, it improved testosterone serum levels in male rats treated with methomyl insecticide [58]. So, vitamin B9 had a beneficial effect in defending against the damage produced by EDCs. Despite the evidence of the benefits of vitamins, the exact mechanisms of how they can protect against the oxidative stress triggered by bisphenols have yet to be discovered. Some reports showed that vitamins do not protect the reproductive system against bisphenol [4]. VitC oxidized cellular components, induced cell death in vitro [33], and lost its efficacy as an antioxidant at high concentrations or partial oxygen pressures. Therefore, in some cases, VitC acts as a pro-oxidant in Sertoli and spermatogenic cells, aggravating the oxidative damage produced by bisphenols [33,59]. VitE was used as a positive control in a study that tested the effects of the echinacoside compound and traditional Chinese medicine cistanche tubulosa as potential agents to ameliorate BPA-induced testicular and sperm damage in rats. Interestingly, it was observed that both echinacoside and cistanche tubulosa had better protection properties than VitE for the reproductive variables affected by BPA administration [41,60].

On the other hand, we reported that VitE produced a synergic harmful effect on diabetic rat pancreas morphology and serum metabolites related to hepatic and renal metabolism when administered with BPS [61]. We observed the same results in healthy rats in our laboratory (manuscript in progress). It is essential to mention that a deficiency of this vitamin in testicular tissue leads to oxidative stress reactions and a decrease in testosterone production and spermatogenesis [47,55]. 

AR and ER are fundamental to the male reproductive system’s function [62]. DHT selectively binds and activates AR in Sertoli cells, initiating spermatogenesis and hindering germ-cell apoptosis [63]. BPA acts as an antagonist by blocking the action of DHT [62,64]. Regarding ER, BPA has a size and shape similar to those of an estradiol molecule; it binds to ER and is less active than human estrogen. However, around 2–3 ppb can induce hormonal activity inside the cytoplasm of cells [7], decrease aromatase enzyme activity, and catalyze the irreversible bioconversion of androgens into estrogens [65]. Bisphenols do not necessarily replicate E2 effects in cells but can initiate distinct, often nonpredictable, nonmonotonic dose responses. So, low doses of bisphenols can contend with natural E2 activity, even in higher circulating E2 concentrations [66]. Our results are consistent with the literature regarding the binding of bisphenols with the AR and ER (Figure 6). However, surprisingly, the VitE and BPS molecular docking results with ER suggest that VitE could interfere or act in combination with BPS and provoke an additive or synergic effect with bisphenol, as we reported [61]. 

Additionally, Khallouki et al. [67] described that VitE is a ligand of ER, demonstrating that VitE acts as a phytoestrogen behaving like a partial agonist in ER-mediated transcriptional regulation of synthetic and endogenous genes. Consequently, to evaluate the effects of VitE, it is crucial to consider that it can behave as an antioxidant and possible EDC. So, we highlight the research opportunity focused on the action mechanism of the vitamins (not only VitE) in combination with bisphenols. Further research on this is advisable, and vitamin administration should be carefully used to guarantee the beneficial effects. 

The antioxidant properties of melatonin could be responsible for the protective effects reported versus the damage produced by bisphenols (acting by their binding to ER [68]) and in many pathologic conditions in the male reproductive system [69]. Also, it has been demonstrated that melatonin stimulates the hypothalamic hypophysis gonadal axis, hence modulating testosterone and estradiol secretion [37,70]. This article’s results indicate that melatonin administration is more effective as a protector against the toxic effects of bisphenol administration than vitamins. Melatonin could act via its antioxidant effect, possibly replacing the action of the antioxidants that are decreased by bisphenol administration and/or through its binding to the hormone receptors. Supporting this last idea, it has been described that melatonin inhibits E2-ER-induced transcription at several ERE-driven promoters through the calmodulin bound to ERα [71] 

Molecular docking examination offers precise data for studying the interaction between a ligand and a protein’s amino acid residues [72]. Docking methods use energy-based scoring to find the energetically most favorable conformation of a ligand bound to a target. Lower energy scores (most negative numbers) indicate preferable protein–ligand bindings compared to higher energy values. MolDock combines a differential evolution optimization technique with a cavity prediction algorithm, using predicted cavities to allow for the fast and precise detection of potential binding modes (poses) [73]. The interaction between the OH phenolic group and Glu353 and Arg394 of ERα is essential for its function. Moreover, VitE had the lowest MolDock score in AR. VitE and VitB9 exhibited the lowest MolDock score in ERα or β. So, the binding of VitB9 and/or VitE with AR, ERα, or ERβ is energetically most favorable compared with the receptor’s natural ligands (E2 and DHT), bisphenols, and VitC. Androgens act through AR, which governs nuclear receptor gene transcription to modulate many biological processes. AR is differentially expressed in many reproductive organs; their signaling at the genomic or the non-genomic level in the testis is essential for spermatogenesis and exerts its effects through testicular Sertoli and peri-tubular myoid cells [74,75]. 

Functional ERs, α and β, are present and have a broad distribution in male reproductive organs throughout development and adulthood. ERs are present in the human fetal testis; they are expressed as early as fetal day 13 in the mesenchyme of the mouse urogenital sinus and on gestational day 16 in the mesenchyme of Wolffian ducts. These ducts form the epididymis, ductus deferens, and seminal vesicles [76,77]. So, fetal male reproductive organs are estrogen targets during their early ambisexual stage and in adulthood. BPS, VitE, and melatonin have a low MolDock score, so the results suggest they could bind to AR and ER α and β. Therefore, it is possible that they could affect the reproductive physiology of mammals exposed to these compounds. So, it is essential to perform studies that evaluate the possible interactions between these ligands and the steroid hormone receptors. Given that vitamins and melatonin interact with AR and ER, we consider it crucial to explore antioxidants that do not display interactions with hormone receptors, such as butylated hydroxytoluene or probucol [67].

Currently, about 20 types of bisphenol analogs are used in daily-use products. According to the results of this systematic review, almost all articles analyzed are focused on BPA, and only one article used octylphenol and nonylphenol. These compounds are also considered ED and are used in the preparation of lubricating oil additives, resins, plasticizers, surface-active agents, detergents, and paints [33] and as a spermicidal agent in condoms and vaginal contraceptives [78]. BPA is present throughout the environment and has been detected in urine, placental tissue samples, amniotic fluid, and blood from umbilical cords taken from individuals living in developed countries [79]. Bisphenol analogs have been detected in saliva, placental tissue, breast milk, and human urine. BPS levels in people from Japan, the United States, China, Kuwait, and Vietnam were 0.933, 0.304, 0.223, 0.126, and 0.148 μg/g creatinine, respectively. Additionally, 0.01 ng/mL BPS and 0.013 ng/mL BPAF have been detected in maternal serum, and 0.03 ng/mL BPS and 0.097 ng/mL BPAF have been identified in cord serum [80,81]. Hence, it is urgent to explore alternative antioxidants that could potentially protect biological organisms against the toxic effects of BPA analogs. 

Finally, we identified some limitations in the present article. First, the limited data in the analyzed reports in this systematic review, such as antioxidant enzymes expressed in the testis and melatonin effects in sperm abnormalities, final body weight, and testis, were insufficient to analyze them statistically. The limited number of data may be because the topic is relatively new, so more experimental studies are needed that use melatonin and vitamins as protector compounds against the noxious effects of bisphenols. Second, the significant heterogeneity of the methods and results, which was demonstrated by *p* < 0.001 in almost all variables (Table 4), is related to the high value obtained for the detection bias (the potential lack of blinding of the outcome assessors). Similar results were observed in a systematic review that evaluated evidence of thermoplastic-type devices linked with the harmful effects of BPA [82]. Therefore, it could be adequate to use guidelines that highlight the relevance of blinding in assessing results. Regarding molecular docking, the results obtained in the present paper should be considered carefully since other studies are needed that experimentally test the interaction between ligands and receptors because the docking accuracy of MolDock is about 87.01% [73]. 

## 4. Materials and Methods

We conducted this systematic review according to the Preferred Reporting Items for Systematic Reviews and Meta-analyses (PRISMA) 2020 guideline [38]. The research question was as follows: Between melatonin and vitamins, which is the most effective compound in protecting rats as an animal model of the harmful effects of bisphenols on reproductive variables? To answer this question, we followed the PECO statement. As Participants (P), we chose male rats; as Exposure, we considered the administration of melatonin or vitamins in addition to bisphenol treatments in the same experiment; as Comparator (C), we selected the control group which was administrated the vehicle of bisphenols; and finally, as Outcomes (O), we selected the effects on reproductive variables as indicators of the protection of melatonin and vitamins against bisphenol actions (spermatic concentration, motility, viability, and abnormalities (head, neck, tail); total, right, and left testis weight; and testosterone). We systematically searched for articles from 1 October 1996 to 12 July 2023, in PubMed first, in Google Scholar second, and finally in Scopus. 

We searched terms, combinations, or similar words as follows: 80-05-7 OR Bisphenol-A OR BPA OR Phenol, 4,4′-(1-methylethylidene)bis- OR Phenol, 4,4′-isopropylidenedi- OR 4,4′-(1-Methylethylidene)bis[phenol] OR 2,2-Bis(p-hydroxyphenyl) propane OR 2,2-Bis(4-hydroxyphenyl)propane OR 80-09-1 OR bisphenol- S OR BPS OR Phenol, 4,4′-sulfonylbis- OR Phenol, 4,4′-sulfonyldi- OR 4,4′-Sulfonylbis[phenol] OR 4,4′-Sulfonyldiphenol OR 4,4′- Dihydroxydiphenyl sulfone OR 79-97-0 OR bisphenol-C OR BPC OR Phenol, 4,4′-(1-methylethylidene)bis[2-methyl]- OR o-Cresol, 4,4′- isopropylidenedi- OR 4,4′-(1-Methylethylidene)bis[2-methylphenol] OR 4,4′-Isopropylidenedi-o-cresol OR 620-92-8 bisphenol-F OR BPF OR Phenol, 4,4′-methylenebis- OR Phenol, 4,4′-methylenedi- OR Phenol, p,p′-methylenedi- OR 4,4′-Methylenebis[phenol] OR Bis(p- hydroxyphenyl)methane OR 1478-61-1 OR bisphenol-AF OR BPAF OR Phenol, 4,4′-[2,2,2-trifluoro-1-(trifluoromethyl)ethylidene]bis- OR Phenol, 4,4′-[trifluoro-1-(trifluoromethyl)ethylidene]di- OR Phenol, 4,4′-[2,2,2- trifluoro-1-(trifluoromethyl)ethylidene]di- OR 4,4′-[2,2,2-Trifluoro-1- (trifluoromethyl)ethylidene]bis[phenol] OR 2,2-Bis(4-hydroxyphenyl) perfluoropropane OR hexafluoro bisphenol A OR 79-94-7OR tetrabromobisphenol-A OR TBBPA OR Phenol, 4,4′-(1-methylethylidene) bis[2,6-dibromo]- OR Phenol, 4,4′-isopropylidenebis[2,6-dibromo]- OR 4,4′-(1-Methylethylidene)bis[2,6-dibromophenol] OR Firemaster BP 4 A OR 2,2-Bis(3,5-dibromo-4-hydroxyphenyl)propane AND Melatonin OR N-acetyl-5-methoxy tryptamine OR n acetyl 5 methoxytryptamine OR/AND Sertoli Cell OR seminal tubules OR fertility OR epididymis OR male reproduction OR testes OR testis OR testicular OR sperm OR Semen OR testosterone OR Seminiferous epithelium AND/OR vitamins, vitamin B (1,2, 3, 5, 6, 7, 9) OR vitamin E OR α,β, *γ, δ* tocopherol OR tocopherols OR tocotrienol OR 5,7,8 trimethyl-tocotrienol.

### 4.1. Study Selection and Eligibility Criteria

All searched articles were scrutinized by reading their title and abstracts in the beginning. Then, we removed duplicate records. The full texts of preliminarily included papers were rescreened to decide the final eligible items. In addition, we manually scoured the reference lists of the documents included in our review to uncover any other relevant citations we may have missed.

The final studies were integrated if they complied with the following criteria: (i) controlled male rat studies; (ii) the subjects were exposed orally or i.p. to bisphenols (BPA, BPS, BPC, BPF, BPAF, tetrabromobisphenol A (TBBPA), nonylphenol, or octylphenol) to induce alterations in reproduction; (iii) at least one intervention group received melatonin or vitamins (B, C, or E) (iv); at least one control group did not receive melatonin or vitamins; (iv) vitamins were administered orally. The exclusion criteria that we took into account were as follows: (i) the study was performed in vitro, ex vivo, or in silico; (ii) the study employed animals that were not the rats; (iii) other endocrine disruptors were studied; (iv) melatonin or vitamins were administered in combination or with other drugs, together at the same time, or melatonin derivatives; (v) the study evaluated other systems or organs such as cardiovascular, digestive, and nervous systems; (vi) the effects were evaluated in the subsequent generations; (vi) reviews, non-English articles, letters, editor letters, congress reports, and clinical trials.

### 4.2. Data Extraction

We used Excel spreadsheets to extract the data and conducted a thorough review to ensure accuracy and to identify errors. The following data were extracted from each study:

1. Article characteristics (first author and publication year).
2. Rat population characteristics (rat strain, total number of animals (intervention, control), weight (g), initial age of exposure (weeks), and age of sample collection (weeks).
3. Antioxidant compound (melatonin or vitamins, dose (mg/kg bw/d), the span of exposure (days), administration route (intraperitoneally: i.p., orally: p.o.).
4. Bisphenol dose (mg/kg bw/d), the span of exposure (days), administration route (i.p. or p.o.)
5. Primary outcomes (reproductive parameters).

### 4.3. Statistical Analysis

We extracted all variables reported by the authors in each article and chose the variables described by two studies with at least three comparisons (two doses or two experimental periods). We excluded data from articles with the same group with very similar results from meta-analysis to avoid bias in the results. We extracted the mean and standard deviation, and we made three comparisons:

1. Data from the bisphenol vs. control group to identify the damage produced by the administration of bisphenols to rats without antioxidant treatment.
2. Data from the bisphenol plus antioxidant (melatonin or vitamins) vs. bisphenol group to determine if the antioxidants restored the adverse effects produced by bisphenol administration alone.
3. Data from the bisphenol plus melatonin or vitamins vs. control group to determine whether administration of antioxidants returns the reproductive variable to control group levels.

We considered that melatonin or vitamins protected reproductive parameters against the toxic effects of bisphenols if no statistical differences were found between the antioxidants plus bisphenol group and the control group and if the result between the antioxidants plus bisphenol group and the bisphenol group was significantly different and closer to the value of the control group.

The statistical data were used for Mean Differences Meta-Analysis using the JAMOVI Software package via the Major module version 2.3. JAMOVI is a free and open statistical spreadsheet built on the R statistical language [83]. A random-effects model was fitted to the data. The Begg test was used to evaluate the publication bias among the studies. Two-tailed *p* values < 0.05 were considered statistically significant. The chi-square test and I^2^ test were used to quantify the heterogeneity between the included studies. When *p* was <0.10 and I^2^ was >50%, we considered heterogeneity to be high; we considered heterogeneity to be low when *p* was >0.10 and I^2^ was <50%. Studies with a studentized residual larger than the 100 × (1 − 0.05/ (2 X k))th percentile of a standard normal distribution were considered potential outliers (using a Bonferroni correction with two-sided alpha = 0.05 for k studies included in the meta-analysis). Studies with a Cook’s distance larger than the median plus 6 times the interquartile range of the Cook’s distances were considered influential. Data were manually extracted from text, tables, figures, or graphs or using Universal Desktop Ruler^®^ software (version 3.6; AVPSoft) when needed.

### 4.4. Study Quality

Two independent reviewers performed the analysis. The systematic evaluation of the bias was assessed using the Systematic Review Center for Laboratory Animal Experimentation (SYRCLE) [83]. This instrument aims to evaluate around ten entries relevant to bias based on five main criteria: selection, performance, detection, attrition, and reporting. A bias rating was established by color: green, low risk; yellow, unclear; and red, high risk. Each item was represented by a number: 1: selection bias, sequence generation; 2: selection bias, baseline characteristics; 3: selection bias, allocation concealment; 4: performance bias, randomization; 5: performance bias, blinding; 6: detection bias, random outcome assessment; 7: detection bias, blinding; 8: attrition bias, incomplete outcome data; 9: reporting bias, selective outcome reporting; 10: other sources of bias [84].

### 4.5. Molecular Docking

To determine whether melatonin or vitamins (E, C, or B9) were ligands for AR or ER and if these molecules could bind the receptors in the same domain as BPA or BPS (we used BPS as a model of a BPA analog), docking studies were performed using the facilities of Molecular Modelling (Molegro Virtual Docker 2007 version 6.0., Molegro Bioinformatics, Aarchus C, Denmark) using E2 and DHT as natural ligand references. We assessed the ligand merged in the crystal structure of the original receptor. Additionally, we considered the interactions between water molecules and the receptor (Table 1). The docking parameters were MolDock Function Score (GRID); grid resolution: 0.30, MolDock SE algorithm; maximum iterations: 1500; maximum size: 50; generating power position threshold: 100; number of poses: 5; intervals: 10–30; number of runs: 10; maximum simplex evolution steps: 300; distance factor: 1.00; energy threshold: 0.00; group raises similar RMSD threshold: 1 (Table 5).

## 5. Conclusions

The results of the present article strongly suggest that melatonin is most effective in preventing the detrimental effects of bisphenols in male reproductive tissue because the vitamins considered cannot prevent the sperm and testosterone concentration decreases produced by bisphenol administration. In contrast, melatonin administration prevented the damaging effects of bisphenols on almost all evaluated variables. Our findings support the current evidence that vitamins can act as EDCs and that their protection against the toxic effects of bisphenols is not the most effective. However, given that vitamins and melatonin interact with AR and ER, we consider it essential to evaluate the effect of antioxidants that do not display interactions with hormone receptors for protection against the harmful toxic effect of bisphenols.

## Figures and Tables

**Table 1:** Characteristics of the included articles.

Rat Strain	Total Animals (Control, Bisphenols, Bisphenol Plus Antioxidants)	Weight (g)	Initial Age of Exposure (Weeks)	Antioxidant Compound	Age at Sample Collection (Weeks)	Reference
**Sprague Dawley**	42 (6, 6, 6)	150–200	8	mel	16–17	Akarca-Dizakar (2020) [24]
**Wistar**	42 (6, 6, 6)	150–170	6	VitC	13	Aydoğan (2010) [33]
**Wistar**	12 (4, 4, 4)	~150–200	7–8	VitC	15–16	Chitra (2003) [4]
**Wistar**	28 (7, 7, 7)	350–400	~11–12	VitB9	~14–15	Gules (2019) [32]
**Sprague Dawley**	42 (6, 6, 6)	190–200	~8	VitE	~14	Jiang (2016) [41]
**Wistar**	24 (6, 6, 6)	231 ± 10	8	VitE	16	Malmir (2021) [42]
**Wistar**	40 (10, 10, 10)	240 ± 10	16	mel	18	Olukole (2018) [37]
**Albino**	30 (6, 6, 6)	150–200	7–8	VitE	15–16	Omran (2017) [43]
**Sprague Dawley**	56 (6, 16, 16)	200–220	8	mel	11–14	Othman (2016) [36]
**Sprague Dawley**	42 (7, 7, 7)	200–220	8	mel VitE	11	Rashad (2021) [44]
**Wistar**	24 (6, 6, 6)	231 ± 10	8	VitE	16	Samira (2020) [40]
**Wistar**	70 (10, 10, 10)	150–200	12	VitE	24	Srivastava (2018) [31]
**Sprague Dawley**	40 (10, 10, 10)	200–220	8	mel	9	Wu (2013) [39]

mel: melatonin; VitB9: vitamin B9 (folic acid); VitC: vitamin C; VitE: vitamin E.

**Table 2:** Details on experimental designs in included articles.

Bisphenol				Antioxidant Compound			Main Outcomes	Reference
BP	Via	Dose *	SE	Via	Dose *	SE		
		**Studies that used melatonin as antioxidant compound**						
**A**	p.o.	25	60	p.o.	20	60	**BPA:** 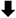 Sperm motility and viability vs. control. **BPA + mel:** 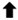 Sperm motility and viability vs. BPA group.	Akarca-Dizakar (2020) [24]
**A**	p.o.	10	14	i.p.	10	14	**BPA:** 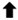 E2 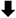 T vs. control, pathological lesions in the prostate. **BPA + mel:** 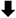 E2 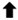 T vs. BPA group. Protector against BPA-induced toxicity in the prostate.	Olukole (2018) [37]
**A**	p.o.	50	21 and 42	p.o.	10	21 and 42	**BPA:** 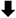 Sperm concentration and motility, T, SOD, and CAT vs. control group. 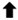 Sperm abnormalities vs. control. **BPA + mel:** 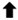 Sperm concentration and motility, T, vs. BPA group.	Othman (2016) [36]
**A**	p.o.	200	10	p.o.	10	10	**BPA:** 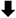 SOD, 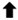 TBARS, sperm DNA damage vs. control group. **BPA + mel:** 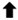 SOD, 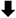 TBARS, and sperm DNA damage vs. BPA group.	Wu (2013) [39]
**Studies that used vitamins as antioxidant compound**								
**A**	p.o.	25	50 ^1^	p.o.	60	50 ^1^	**BPA, NP, OP:** No alterations in testicles and epididymis absolute weight vs. control group. 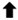 pathological lesions in testicles vs. the control group. **BPA + VitC, NP + VitC, OP + VitC:** 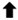 Histopathological lesions in testicles vs. control group. 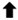 Relative epididymis weight	Aydoğan (2009) [33]
**NP**	p.o.	25		p.o.	60			
**OP**	p.o.	25		p.o.	60			
**A**	p.o.	0.0002, 0.002, 0.02	60	p.o.	40	60	**BPA:** 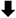 Epididymal sperm motility and concentration, antioxidant enzymes, equal sperm viability compared to control group. **BPA + VitC:** 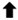 Epididymal sperm motility and concentration, antioxidant enzymes vs. BPA group.	Chitra (2003) [4]
**A**	p.o.	50	14	p.o.	20	14	**BPA:** 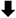 Seminiferous tubule height and T. 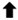 TUNEL-positive cells per seminiferous tubule. **BPA + VitB9:** Seminiferous tubule height, T and TUNEL-positive cells equal to control.	Gules (2019) [32]
**A**	p.o.	200	42	p.o.	300	42	**BPA:** 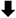 Sperm concentration, semen production, T, LH vs control group. **BPA + VitE:** No differences in sperm variables, abnormal tail, and FSH vs. control and BPA groups.	Jiang (2016) [41]
**A**	p.o.	250	56	p.o.	150	56	**BPA:** 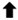 TUNEL-positive spermatocytes, spermatids, and Sertoli cells vs. control group. 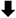 T, motility, concentration, viability vs. control group. Body weight is equal to the control group. **BPA + VitE:** TUNEL-positive spermatic cells, body weight, and T similar to control group.	Malmir (2021) [42]
**A**	p.o.	325	56	p.o.	200	56	**BPA:** 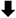 T, 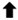 sloughing of the germinal epithelium vs. control. **BPA + VitE:** 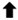 T, 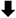 Spermatogonia number, and mild sloughing in germinal epithelia vs. BPA group. Disruption of germinal epithelium.	Omran (2017) [43]
**A**	i.p.	50	21 ^1^	p.o.	VitE (100)	21 ^1^	**BPA:** 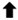 Abnormal sperm morphology vs control. 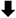 Sperm concentration and motility, T vs. control group. **BPA + VitE o BPA + mel:** 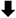 Abnormal spermatozoa vs. BPA group. 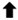 Sperm concentration and motility, T vs. BPA group.	Rashad (2021) [44]
				i.p.	mel (10)			
**A**	p.o.	250	56	p.o.	150	56	**BPA:** 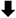 Seminiferous tubule diameter, germinal epithelium thickness, number of spermatocytes, spermatids, Sertoli cells, sperm concentration, and T vs. control group. **BPA + VitE:** equal T vs. control group. 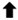 seminiferous tubule diameter, germinal epithelium thickness, spermatocyte, sperm concentration vs. BPA group.	Samira (2020) [40]
**A**	p.o.	0.05, 0.5, 1	90	p.o.	40	90	**BPA** (high dose): 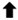 Epididymis apoptosis, 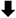 T vs. control group. **BPA + VitE:** 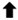 Testis weight vs. BPA group.	Srivastava (2018) [31]

BP: bisphenol; BPA: bisphenol A; CAT: catalase; E2: estradiol; FA: folic acid; FSH: follicle-stimulating hormone; GSH-Px: glutathione peroxidase; i.p.: intraperitoneally; MDA: malondialdehyde; mel: melatonin; NP: nonylphenol; OP: octylphenol; p.o.: per oral; SOD: superoxide dismutase; SE: span of exposure (days); T: testosterone serum concentration; TBARS: thiobarbituric acid reactive substances; TUNEL: terminal deoxynucleotidyl transferase (TdT) dUTP nick-end labeling assay; VitB9: vitamin B9 (folic acid); VitC: vitamin C; VitE: vitamin E; * (mg/kg bw/d); 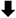 decrease; 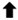 increase; ^1^ three times each week.

**Table 3:** Meta-analysis outcomes on final body weight, sperm characteristics, hormonal parameters, and testis weight.

Analysis	SMD	95% CI	Heterogeneity			*p*	Begg Test	Egger Test	No. Studies (No. Comparisons)
			I^2^ (%)	*p* Het	*Q*				
**Final Body Weight**									
**BP vs. Ctrl ^2^**	−0.65	−2.38, 1.07	89.35	<0.001	22.5	0.380	0.817	<0.001	3(5)
**BP + vit vs. BP**	−0.63	−2.68, 1.42	91.1	<0.001	37.4	0.547	0.233	0.430	3(5)
**BP + vit vs. Ctrl**	−1.07	−1.61, −0.53	0.00	0.436	3.8	<0.001 *	0.083	0.105	3(5)
**Testosterone serum concentration**									
**BP vs. Ctrl ^1^**	−9.37	−12.66, −6.08	77.56	0.002	12.5	<0.001 *	0.083	<0.001	3(4)
**BP + mel vs. BP**	6.34	3.92, 8.77	82.87	<0.001	25.1	< 0.001 *	1.000	0.007	3(4)
**BP + mel vs. Ctrl**	−2.53	−5.65, 0.60	95.78	< 0.001	39.8	0.113	0.083	<0.001	3(4)
**BP vs. Ctrl ^2^**	−3.68	−6.10, −1.26	95.73	<0.001	56.5	0.003 *	0.006	< 0.001	6(8)
**BP + vit vs. BP**	1.61	0.32, 2.91	89.72	<0.001	31.5	0.015 *	0.002	<0 .001	6(8)
**BP + vit vs. Ctrl**	−2.08	−3.83, −0.32	93.74	<0.001	46.9	0.021 *	0.075	< 0.001	6(8)
**Sperm concentration**									
**BP vs. Ctrl ^1^**	−3.82	−7.16, −0.47	95.32	< 0.001	46.2	0.025 *	0.017	<0.001	4(5)
**BP + mel vs. BP**	2.17	0.05, 4.30,	91.68	< 0.001	32.1	0.045 *	0.017	<0.001	4(5)
**BP + mel vs. Ctrl**	−0.77	−2.71, 1.16	92.04	<0.001	27.5	0.434	0.483	<0.200	4(5)
**BP vs. Ctrl ^2^**	−2.10	−3.01, −1.19	83.18	<0.001	54.3	0.001 *	0.006	<0.001	6(14)
**BP + vit vs. BP**	1.76	0.98, 2.53	79.15	<0.001	50.0	<0.001 *	< 0.001	<0.001	6(14)
**BP + vit vs. Ctrl**	−1.23	−1.86, −0.60	71.38	<0.001	42.2	<0.001 *	0.101	0.045	6(14)
**Sperm motility**									
**BP vs. Ctrl ^1^**	−10.85	−18.78, −2.93	95.47	<0.001	82.1	0.007 *	0.083	<0.001	3(4)
**BP + mel vs. BP**	6.11	−0.72, 12.94	98.69	<0.001	50.1	0.079	0.750	<0.001	3(4)
**BP + mel vs. Ctrl**	0.46	−3.71, 4.63	97.10	<0.001	53.1	0.827	1.000	0.751	3(4)
**BP vs. Ctrl ^2^**	−11.10	−13.71, −8.49	0.00	0.583	1.95	<0.001 *	0.083	0.170	2(4)
**BP + vit vs. BP**	13.42	5.32, 21.52	84.51	<0.001	22.5	0.001	0.083	<0.001	2(4)
**BP + vit vs. Ctrl**	−1.23	−3.19, 0.73	85.08	0.001	15.6	0.218	0.083	<0.001	2(4)
**Sperm viability**									
**BP vs. Ctrl ^1^**	−2.58	−4.29, −0.88	79.95	0.005	10.5	0.003 *	0.333	0.001	2(3)
**BP + mel vs. BP**	5.27	1.03, 9.50	94.73	<0.001	46.0	<0.001 *	0.333	<0.001	2(3)
**BP + mel vs. Ctrl**	−2.58	−4.29, −0.88	79.95	0.005	10.5	0.003 *	0.333	0.001	2(3)
**BP vs. Ctrl ^2^**	−2.79	−6.23, 0.66	93.49	<0.001	19.3	0.113	0.083	<0.001	2(4)
**BP + vit vs. BP**	1.83	−0.42, 4.09	87.24	0.001	16.1	0.111	0.083	<0.001	2(4)
**BP + vit vs. Ctrl**	−0.93	−1.75, −0.11	29.08	0.254	4.1	0.026 *	1.000	0.201	2(4)
**Abnormal sperm morphology**									
**BP vs. Ctrl ^2^**	4.99	1.92, 8.06	91.21	<0.001	30.1	0.001 *	0.017	<0.001	3(5)
**BP + vit vs. BP**	−1.43	−4.26, 1.40	94.43	<0.001	47.4	0.321	0.483	0.166	3(5)
**BP + vit vs. Ctrl**	5.06	2.75, 7.36	81.86	<0.001	21.3	<0.001 *	0.017	<0.001	3(5)
**Abnormal sperm head morphology**									
**BP vs. Ctrl ^2^**	1.55	−0.73, 3.83	90.7	<0.001	26.2	0.184	0.333	<0.001	2(4)
**BP + vit vs. BP**	0.63	−3.68, 4.93	95.9	<0.001	50.8	0.775	1.000	0.736	2(4)
**BP + vit vs. Ctrl**	2.17	0.17, 4.18	85.58	<0.001	26.6	0.034 *	0.083	<0.001	2(4)
**Abnormal sperm neck morphology**									
**BP vs. Ctrl ^2^**	3.50	−0.30, 7.30	95.64	<0.001	17.9	0.071	0.083	<0.001	2(4)
**BP + vit vs. BP**	−1.31	−3.89, 1.28	92.61	<0.001	29.4	0.321	0.750	0.066	2(4)
**BP + vit vs. Ctrl**	2.68	0.40, 4.96	88.35	<0.001	19.8	0.021 *	0.083	<0.001	2(4)
**Abnormal sperm tail morphology**									
**BP vs. Ctrl ^2^**	2.49	−0.08, 5.06	91.73	<0.001	18.5	0.058	0.083	<0.001	2(4)
**BP + vit vs. BP**	−0.15	−1.09, 0.78	61.37	0.054	7.6	0.746	0.750	0.171	2(4)
**BP + vit vs. Ctrl**	3.64	−0.11, 7.39	94.35	<0.001	36.1	0.057	0.083	<0.001	2(4)
**Total testis weight**									
**BP vs. Ctrl ^2^**	−1.73	−2.87, −0.59	79.34	<0.001	18.9	0.003 *	0.233	0.104	3(5)
**BP + vit vs. BP**	1.42	0.17, 2.67	83.46	<0.001	23.8	0.026 *	0.817	0.428	3(5)
**BP + vit vs. Ctrl**	−0.70	−1.14, −0.26	0.00	0.803	1.6	0.002 *	0.483	0.284	3(5)
**Right testis weight**									
**BP vs. Ctrl ^2^**	−0.81	−1.84, 0.21	65.24	0.039	8.39	0.119	0.083	0.005	2(4)
**BP + vit vs. BP**	−0.13	−1.01, 0.76	57.43	0.071	7.03	0.778	0.333	0.555	2(4)
**BP + vit vs. Ctrl**	−1.57	−2.50, −0.63	50.80	0.106	6.13	<0.001 *	0.083	0.013	2(4)
**Left testis weight**									
**BP vs. Ctrl ^2^**	−0.49	−1.44, 0.45	62.22	0.054	7.64	0.304	0.333	0.012	2(4)
**BP + vit vs. BP**	−0.19	−0.97, 0.59	46.31	0.133	5.60	0.634	0.750	0.609	2(4)
**BP + vit vs. Ctrl**	−0.79	−1.37, −0.22	0.00	0.938	0.41	0.007 *	0.083	0.524	2(4)

BP: bisphenol; CI: confidence interval; Ctrl: control; I^2^: a statistic estimating fraction of variance due to heterogeneity of studies; mel: melatonin; P het: *p* value of heterogeneity of studies; Q: Cochran’s test, a measure of heterogeneity; SMD: standardized mean difference; vit: vitamins; ^1^ studies performed with melatonin; ^2^ studies performed with vitamins. *: *p* < 0.05.

**Table 4:** Types of interaction between receptor’s amino acids and ligands.

Receptor	Ligand	Amino Acids with Hydrogen Bonds	Amino Acids with Steric Interaction	Functional Groups and Attached Amino Acids	MolDock Score
**Androgen**	E2	Arg 752, Thr 877, Gln 711	Met 745	OH (phenol) -> Gln 711, Arg 752, Met 745 OH (cyclopentane) -> Thr 877	−117.344
	DHT	Arg752, Thr 877, Gln711	Met 745, Phe 764	OH (carbonyl) -> Gln 711, Arg 752 OH (cyclopentane) -> Thr 877	−109.764
	BPA	Arg 752, Gln 711, Asn 705, Phe 764	-	OH (phenol) -> Arg 752, Gln 711, Phe 764, Asn 705	−90.4377
	BPS	Arg 752, Gln 711, Asn 705	Thr 877	OH (phenol) -> Gln 711, Arg 752, Asn 705	−91.184
	mel	Met 745	Leu 704	Pyrrole -> Met 745	−114.947
	VitE	Thr 877	Met 787, Asn 705, Leu 880, Thr 877, Phe 876, Leu 873, Met 745, Phe 764, Leu 704, Met 895, Gly 708, Arg 752, Met 749, Gln 711, Val 746	OH (phenol) -> Thr 877 Phenol -> Asn 705, Met 895, Leu 704	−139.586
	VitC	Arg 752, Gln 711, Leu 704, Met 745	Gly 708, Met 749	Carbonyl -> Gln 711, Arg 752, Phe 764 OH-3 -> Met 745, Met 749 OH-6/7-> Leu 704	−97.1359
	VitB9	Leu 701, Thr 877, Asn 705, Leu 873	Leu 873, Phe 876, Met 780, Leu 704, Gly 708, Met 742, Val 746, Met 749, Met 787, Met 745	NH2 (cyclohexene): Met 787, Met 749 Carbonyl -> Met 742, Met 745 NH (cyclohexene): Val 746 Phenol: Leu 704	−136.277
**Estrogens** **(α chain)**	E2	Arg 394, His 524, Glu 353	-	OH (phenol) -> Glu 353, Arg 394 OH (cyclopentane) -> His 524	−102.505
	DHT	Arg 394, His 524, Gly 521	Leu 387, Glu 353, Leu 525	OH (cyclopentane) -> His 524, Gly 521 OH (carbonyle) -> Glu 353, Arg 394	−101.016
	BPA	Thr 347, Phe 404	Arg 394, Glu 353, Leu 384	OH (phenol) -> Phe 404, Glu 353, Thr 347	−83.136
	BPS	Phe 404	Arg 394, Glu 353, Phe 404, Met 343, Thr 347, Leu 349, Leu 384, Met 384, Met 388	OH (phenol) -> Leu 349, Phe 404 Sulfoxide -> Met 388, Leu 384	−82.0316
	mel	-	Leu 391, Glu 353	Phenol-> Leu 346	−100.315
	VitE	Glu 353	Asp 351, Leu 428, Met 388, Leu 387, Leu 391, Glu 353, Leu 346, Phe 404, Met 343, Leu 349, Leu 384, Leu 525, Gly 521, His 524, Ile 424	OH (phenol) -> Leu 391, Glu 349	−128.716
	VitC	Arg 394, Glu 353, Leu 387	Leu 346, Glu 353, Leu 391, Met 388, Phe 404	Carbonyl -> Leu 346 OH-2: Glu 353, Phe 404 OH-3: Leu 387, Leu 391, Arg 394 OH-5: Met 388	−72.1521
	VitB9	Leu 387, Glu 353, Asp 351	Leu 387, Arg 394, Lys 529, Thr 347, Ala 350, Trp 383	NH2 (cyclohexene): Leu 387, Glu 353, Arg 394, Carbonyl -> Lys 529, Thr 347, Trp 383	−136,387
**Estrogens ** **(β chain)**	E2	Arg 346, Glu 260	Leu 343	OH (phenol) -> Glu 305, Arg 346	−100.014
	DHT	Arg 346	Leu 343, Leu339, Gly472, Glu 305, Leu 476	Carbonyl -> Arg 346, Glu 305	−111.186
	BPA	Arg 346, Glu 305	Phe 356, Met 336	OH (phenol) -> Glu 305, Arg 346	−92.5984
	BPS	Arg 346, Glu 305	Phe 356, Met 336, Leu 339	OH (phenol) -> Glu 305, Arg 346 O (sulfoxide) -> Leu 339, Met 336	−80.4489
	mel	Glu 305, Leu 298	Leu 343	N (secondary amine) -> Leu 298 O (Ether) -> Leu 343 N (pyrrole) -> Glu 305	−105.784
	VitE	Leu 398	Ile 373, Met 340, Leu 298	OH (phenol) -> Leu 398	−148.873
	VitC	Arg 346, Leu 298, Leu 339	Leu 343, Phe 356, Ala 302	Carbonyl -> Arg 346, Phe 356 OH-3: Leu 339, Leu 343 OH -> 6/7: Leu 298	−98.4997
	VitB9	Asp 303, Pro 486, Glu 305	Arg 346, Ala 302, Trp 335, Leu 306, Leu 476, Pro 486	NH2 -> Glu 305 OH -> Pro 486, Asp 303	−150.355

BPA: bisphenol A; BPS: bisphenol S; DHT: dihydrotestosterone; E2: estradiol; mel: melatonin; VitC: vitamin C; VitB9: vitamin B9; VitE: vitamin E; -: no data obtained; -> join.

**Table 5:** General parameters for molecular docking.

Receptor	Coordinates	Radio Å	PDB Code
**Androgen**	X: 0.41, Y: 31.66, Z: 4.44	10	1E3G
**Estrogens (α chain)**	X:90.42, Y: 13.12, Z: 72.44,	10	1NDE
**Estrogens (β chain)**	X: 109.00, Y: 8.82, Z: −108.68	10	1A52

PDB: Protein Data Bank.

## Data Availability

Data are available from the corresponding author upon reasonable request.
